# Qualitative Evaluation of a Vicarious Trauma e-learning Initiative for Cancer Support Line Workers in the UK

**DOI:** 10.1007/s13187-025-02674-3

**Published:** 2025-06-27

**Authors:** Donna Munro, Andrew Killen, Karen Campbell

**Affiliations:** 1 Royal College of Nursing, London, England; 2 University of the West of Scotland, Glasgow, Scotland; 3 Edinburgh Napier University, Edinburgh, Scotland

**Keywords:** Vicarious trauma, E-learning, Cancer support line

## Abstract

The concept of vicarious traumatisation (VT) is distinguished from psychological stress concepts of ‘burnout’, ‘compassion fatigue’, and ‘secondary traumatic stress’. Cancer support line workers may have a heightened risk of VT. Taking education as an intervention for VT, the aim of this study was to capture pre- and post-perceptions of an e-learning intervention for cancer support line workers. To explore cancer support line workers’ perceptions of VT pre- and post- e-learning intervention. Ten study participants were surveyed prior to a VT e-learning intervention. Nine study participants completed e-learning, followed by semi-structured interviews. Reflexive thematic analysis was utilised. Five themes emerged: pre-existing understanding of VT, resonance, recognition, refocus self-care, and reflection on the impact of VT experience. Varied levels of understanding of the concept of VT were identified. The module was impactful and increased understanding of VT for most participants. A requirement for formalised VT education, subsequent signposting, and support, was identified. Participants expressed desire for refresher courses.

## Introduction

McCann and Pearlman [1] define vicarious trauma as the emotional and psychological impact that trauma workers experience as a result of empathising with and hearing about the traumatic experiences of others. It occurs when individuals are repeatedly exposed to the trauma narratives of others, which can lead to changes in their own worldview, sense of safety, trust, and personal beliefs. There are key differences between the other concepts of secondary trauma and VT, where VT is associated with empathetic engagement in combination with secondary trauma [1–3]. Workers are familiar with concepts such as ‘burnout’ and ‘compassion fatigue’; however, vicarious trauma is distinguished from these due to the cognitive reaction it fuels [1].

Vicarious trauma involves shifts in how a person perceives the world, often resulting in symptoms such as emotional numbing, intrusive thoughts, or feelings of hopelessness. McCann and Pearlman [1] emphasise that this process is cumulative, with the impact becoming more pronounced over time as workers are exposed to increasingly distressing stories.

Wu et al. [2] extends the concept of VT specifically to nursing, describing the multifaceted nature of VT and the impact on nurse’s cognitive schema from clinical practice from deeply empathising with the physical or emotional trauma of patients, families, and colleges. Furthermore, there are both internal and external factors at play [2]. Internal factors include personal psychological characteristics [4], trauma history [5], and those with a high empathetic engagement [6] attending to their own wellbeing [7]. External factors include repeated dealings with trauma from others illness and death [8] and insufficient organisational support [9].

Therefore, understanding vicarious trauma is critical due to its far-reaching implications for individual well-being, workplace performance, and the overall effectiveness of caregiving and support systems [2]. Despite growing awareness, many organisations lack adequate strategies to identify, mitigate, and manage its impact [2].

Interventions for vicarious trauma (VT) typically focus on supporting individuals who are experiencing the emotional and psychological effects of repeated exposure to trauma, fostering resilience, and preventing further harm. Some effective interventions include supervision and peer support, self-care practices, boundary setting; therapy and counselling, workplace policies and organisational support and training and education [10, 11].

Although VT has been studied extensively in other professions [12], with limited application to oncology nursing [13], there is a need to apply this knowledge to the growing profession of being a cancer support line worker, developed to cater for the growing cancer population, with information and emotional needs.

The literature, however, is sparse in regard to interventions available specifically related to cancer support line workers. A support line worker may have to manage high-stress situations, often dealing with callers who are in crisis or distress. They might work in a fast-paced environment with the need to remain calm, empathetic, and focused. The job may require handling multiple calls in succession, often without breaks, whilst maintaining confidentiality and privacy. Additionally, they may work during nights, weekends, and holidays, depending on the hotline’s availability, and may be exposed to emotional or traumatic content that requires mental resilience. Willems et al. [14] exploration of the emotional challenges faced by crisis line volunteers, concluding that access to training would help cope with traumatic calls. Currently, there is little focus on how to educate workers on the risks [12, 15, 16].

Taking education as an intervention for VT, the aim of this study was to capture pre- and post-perceptions of an e-learning intervention for cancer support line workers.

### Method

#### Design

This study adopted a qualitative design to evaluate an e-learning educational intervention [17].

#### Participants

Participants were purposively sampled from those currently working on the cancer support line.

#### Ethical Considerations.

Ethical approval was obtained from the University of the West of Scotland, Ethics Committee. Additionally, Macmillan Cancer Support, Directorate of Direct Services, granted permission for service evaluation.

### Learning Objects

Learning objects were delivered through an easily accessible digital platform. Emotive professional experience videos were utilised. Theoretical content provided essential knowledge on recognising signs and symptoms of (VT), improving understanding and awareness.

Interactive elements like personality trait questionnaires helped learners explore personal drivers and coping strategies. Reflective activities encouraged self-care and introspection. Participants expressed a desire for more time dedicated to reflection.

### Data Collection

Ten support line workers were invited to participate and nine agreed to participate. Informed written consent was obtained from study participants 24 h after viewing the participant information sheet and signing the consent form. Semi-structured interviews were conducted lasting 30 to 45 min using a semi-structured questions format [17]. Thematic data saturation occurred when semi-structured interviews produced no further novel insights came from the semi- structured interviews [18].

### Data Analysis

Two researchers (KC and DM) completed a six-step method of thematic analysis with coding of specific themes [18]. This involved identification of patterns and themes present in the qualitative data, points that appeared pertinent to the subject of VT and took cognisance of the difference between semantic, explicitly clear themes and latent themes, which had an underlying context [19]. The study’s rigour was reinforced using multiple coders in the analyses [18]. A continuous, reflexive approach was used by the two independent researchers during coding by questioning interpretation whilst analytically working through themes [18]. Both researchers’ prior knowledge on the subject was evidenced in the identification of latent themes, although some were semantically defined within transcripts.

## Findings

The findings are presented in five themes: *Pre-existing understanding of VT*,* Recognition*, *Resonance*, *Refocus self-care*,and* Reflection on the impact of VT experience. Pre-existing understanding of VT* was categorised into four levels of understanding Table 1).

**Table 1:** The four levels associated with pre-existing understanding of VT before the e-learning module

**Level of understanding**	**Number of participants**
1. No understanding: participants had never received information on, or read about VT	Four
2. Minimal understanding: participants were able to partially explain the impact of VT	Three
3. Moderate understanding: participants were able to define that ‘repeated’ or ‘constant’ exposure to other people’s trauma was an intrinsic element of Vicarious Traumatisation	Two
4. Extensive understanding: participants were able to fully define the concept of VT, describe the impact VT may have on an individual and explain how to mitigate risk of VT	Zero

### Recognition

As participants moved through the eLearning, they recognised elements of VT within other concepts of Burnout and Compassion Fatigue*.* They identified a fundamental difference between VT and other concepts that it links directly to feeling the trauma of others. Participants were subsequently able to provide in-depth descriptions of VT. One participant stated the module had allowed them to appreciate the difference and physical effects on the body:

I understand much better the impact, not just of empathising with people, but of just absorbing that trauma because I didn’t really think of it in that way before. I just thought, you know, you just got tired with things.

### Resonance

Learning objects such as videos afforded opportunities to view the ‘ Vicarious role’ which collectively resonated with all nine participants. This was in relation to the impact of their role on their physical and emotional health. This resonance resulted in them sharing reflections on their own vicarious roles, within the interviews, and the impact on their physical and mental health. One participant described:

I felt like it summed up [the air ambulance doctor experience] the context and the journey that most of us go through at some point really well and how to be able to then come back from that and use that as a learning experience to improve ourselves and us professionally as well.

Resonance was felt with the pressures of having a continuous flow of traumatised people to deal with and how this could take its toll. One participant described that this continuous flow in their role as a specialist support line professional.

Quite often you can just sort of go oh I’ll just take one more call. I’ll just take one more call. It’s quite busy today and you’ll maybe push yourself further than you should rather than taking a break.

### Refocus on Self-care

A couple of participants suggested there may be a ‘shelf-life’ to their role, prior to undertaking the module. On completion, this perception changed, with participants recognising that education on VT helped them understand the impact of their role and how they could re-ground or refocus themselves.

All participants, except for one, identified consistency in practising self-care, which was challenging.

The module allowed contemplation of self-care and participants admitted the module was a good reminder, an aid to self-care. They evidenced this in descriptions of practical self-care techniques which had been taught within the module, grounding energy, releasing negative energy, and recharging positive energy. The techniques were identified as ‘new’ and participants previously had not considered them to be a way of mitigating risk of VT.

One participant stated: ‘I do the exercises… …it loosens everything up actually, it works.’

### Reflection on Impact of VT Experience

Inevitably, the module content enabled and stimulated participants to reflect on their own experiences of VT (Table 2).

**Table 2:** The participants own experiences of past and present VT

Experience of VT	Additional context
Suffering from VT at time of interview	Work related
Suffering from VT at time of interview	Related to caring responsibilities at home, not work
Suffering from VT at time of interview as well as having suffered from VT in the past	Described symptoms as ‘creeping in’ at present—has caring responsibilities at work and at home Identified with suffering from VT in current work role as well as within a previous work role
Suffered from VT very recently, feels as though moving out of acute phase of VT now. Has also suffered from VT in the past	Work related though has at times had caring responsibilities at home in addition to work. Identified with suffering from VT in current work role as well as within a previous work role
Suffered from VT in the past	Work related—whilst in current role
Suffered from VT in the past	Work related—in a previous role
Suffered from VT in the past	Work related—whilst in current role
Has never had VT	No additional context
Has never had VT	Has suffered from ‘Burnout’

The module was described as ‘really impactful’ and ‘empowering’ by Participant A. This sentiment was replicated amongst other participants through memories of past experiences, providing clarity in relation to past experiences. As participants reflected on suffering from VT, they realised that they had not understood what it was. Completing the module provided a point of realisation, helping them understand their suffering and why it had occurred. This was reflected in a participant recollection:

…I could relate to everything that was in the course because I thought that’s what was wrong with you and there was no help. There was no help for the helper. You just shut off. You feel stupid. It’s being able to recognise it and realise that what you’re feeling is valid because you feel that you’re absolutely alone. You feel you’re the only one, you’re the idiot. You’re the one that’s like not coping, you know. And that’s what the course does. It helps you recognise it and gives you a solution.

Participants were able to reflect that during acute episodes of VT, their behaviour had been different, less empathic, and not in keeping with their natural behaviour. Participants were then able to make the link, that once one has reached an acute episode of VT, it then becomes challenging to look after wellbeing and fulfil your working role to maximum potential.

One participant hypothesised that one’s own lived experiences were more strongly associated with a risk of VT, and risk was increased if the person they were interacting with had an experience that mirrored their own. They felt this was more significant than the neurophysiology and said:

Because I’ve gone through that experience, my empathy is completely changed, and I find myself more engaged with the person because they’ll turn round and say that is exactly what it’s like. So that affects your vicarious trauma because you’re actually reliving it.

Having more in-depth knowledge led participants to think about risk mitigation in terms of self-care as opposed to reaching a point where they may struggle. This was important as there was a consensus from individuals that VT can develop gradually and almost subconsciously. A participant shared:

It really put the focus on the importance of wellbeing and just finding those little moments in what’s a really busy schedule for most people…

## Discussion

This study is a unique account of an exploration of the perceptions of cancer support line workers in relation to VT. The prior level of understanding of VT was varied amongst participants. Upon completion of the module, these findings show a shift with the new learning, where the level of understanding now mirrors other literature defining [1–3].

It was apparent from the participants describing reliving trauma that they had engaged deeply in the e-learning content. They recognised that repeated exposure of consistently empathising and absorbing the trauma of others in conjunction with the repetitive nature of the back-to-back calls were contributory factors to VT [2]. This shift in learning was enhanced though the delivery of emotive video content, with tangible examples of day-to-day practice. This is in keeping with the theory of authentic learning, where participants were more likely to apply their learning when they returned to their roles [20].

Self-reflection in relation to the drivers/personality traits was stimulated by the module. Participants judged the impact of their own drivers, during e-learning exercises, on whether they impacted their risk of VT. Costa and Moss [21] emphasised the need for increased awareness of psychological symptoms of distress in order to retain the workforce. This would imply that attrition rates of support line workers can be affected by education on the impact of their role and how to cope with the impact.

Although participants could historically see the value of self-care, they continued to put others before themselves. Allocating a specific time for self-care out-with work seemed to be a solution for some, with participants finding if they did not do this, then self-care did not happen. There were similar findings in the study by Roberts et al. [16], whereby employees valued self-care and used a variety of activities to improve psychological wellbeing.

Similarities can be drawn between the findings of the current study amongst support line workers and the findings by Willems et al. [14], amongst crisis line workers, where they concluded that personal resources and self-awareness are intrinsic to suitably managing one’s own emotions, and the current study displays how insightful it was for participants to explore their own personal drivers and coping mechanisms through the use of the module learning objects.

It has been identified that both the internal factors and external factors associated with the cancer support worker role will increase the likelihood of exposure to VT. Providing vicarious trauma training is critical to recruiting and retaining support line workers, as it equips them to manage the emotional toll of their work and reinforces an organisational culture, through policy, that prioritises their mental health and resilience. Future studies could explore the connection between the type of personalities attracted to the roles and how this affects their risk of VT. Taking cognisance of the impact of racialised trauma [22] is also important for future studies, to explore whether those who have suffered from racialised trauma are at higher risk of VT.

## Conclusion

The VT e-learning showed that providing education helps employees to realise their risk, recognise their own experiences, past or present, and decrease that risk by implementing the suggested strategies. The study found workers in vicarious roles feeling overwhelmed at times, strengthening the support for more widespread education to support risk mitigation of VT. Future research must include testing the e-learning course in other settings and with a larger sample of participants. The future research should also include a defined educational framework to support the evaluation to enhance the knowledge about behavioural changes and reduction in VT symptoms beyond self-reported perceptions.

### Limitations

There are a number of limitations to the study. There may have been self-selection bias introduced, through attracting people that had an existing interest in the topic. Generalisability is limited due to the small sample size; nevertheless, the findings give insight into the important patterns that warrant further investigation. Future research into the impact of vicarious traumatisation on a broader range of helpline cancer professionals could help to define the scale of the issue and need to mitigate risk. In addition, this study gave insight into important e-learning on VT that could be tested with greater numbers and other workforce areas. This evaluation did not directly monitor engagement.

## References

[1] McCann IL, Pearlman LA (1990) Vicarious traumatization: a framework for understanding the psychological effects of working with victims. J Trauma Stress 3(1):131–149. 10.1002/jts.2490030110

[2] Wu Y, Bo E, Yang E, Mao Y, Wang Q, Cao H, He X, Yang H, Li Y (2024) Vicarious trauma in nursing: a hybrid concept analysis. J Clin Nurs 33(2):724–739 10.1111/jocn.16918 37926935 10.1111/jocn.16918

[3] Kennedy S, Booth R (2022) Vicarious trauma in nursing professionals: a concept analysis. Nurs Forum 57:893–897 10.1111/nuf.12734 35478459 10.1111/nuf.12734

[4] Isobel S, Thomas M (2022) Vicarious trauma and nursing: An integrative review. Int J Mental Health Nurs 31:247–259. 10.1111/inm.12953 10.1111/inm.12953 34799962

[5] Yu H, Jiang A, Shen J (2016) Prevalence and predictors of compassion fatigue, burnout and compassion satisfaction among oncology nurses: a cross-sectional survey. Int J Nurs Stud 57:28–38 10.1016/j.ijnurstu.2016.01.012 27045562 10.1016/j.ijnurstu.2016.01.012

[6] Oğlak SC and Obut M (2020) The risk of vicarious trauma among front-line and non-front-line midwives and nurses: vicarious traumatization among medical staff. Aegean J Obstet Gynecol 2(2):19–22 10.46328/aejog.v2i2.40

[7] Norhayati MN, Che Yusof R, Azman MY (2021) Vicarious traumatization in healthcare providers in response to COVID-19 pandemic in Kelantan. Malaysia PLoS One 16(6):e0252603 10.1371/journal.pone.0252603 34086747 10.1371/journal.pone.0252603 PMC8177457

[8] Wies J, and Coy, K (2013) Measuring violence: vicarious trauma among sexual assault nurse examiners. Hum Organ 72(1):23–30 10.17730/humo.72.1.x5658p957k5g7722

[9] Wang N, Liao K, Zheng W, Zhou T, Sun S, Yang Y, Xu J (2022) Correlation between coping styles of clinical nurses and vicarious trauma under the normalization of epidemic prevention and control. Nursing Practice and Research 19(24):3665–3670 10.3969/j.issn.1672-9676.2022.24.007

[10] Bercier ML, Maynard BR (2015) Interventions for secondary traumatic stress with mental health workers: a systematic review. Res Soc Work Pract 25(1):81–89 10.1177/1049731513517142[9]

[11] Kim J, Chesworth B, Franchino-Olsen H, Macy RJ (2022) A scoping review of vicarious trauma interventions for service providers working with people who have experienced traumatic events. Trauma Violence Abuse 23(5):1437–1460 10.1177/1524838021991310 33685294 10.1177/1524838021991310 PMC8426417

[12] Molnar BE, Sprang G, Killian KD, Gottfried R, Emery V, Bride BE (2017) Advancing science and practice for vicarious traumatization/secondary traumatic stress: a research agenda. Traumatology 23(2):129–142 10.1037/trm0000122

[13] Sinclair HA, Hamill C (2007) Does vicarious traumatisation affect oncology nurses? A literature review. Eur J Oncol Nurs 11(4):348–356. 10.1016/j.ejon.2007.02.007 17482879 10.1016/j.ejon.2007.02.007

[14] Willems R, Drossaert C, Vuijk P, Bohlmeijer E (2021) Mental wellbeing in crisis line volunteers: understanding emotional impact of the work, challenges and resources. A qualitative study, International Journal of Qualitative Studies on Health and Well-being 16(1) 10.1080/17482631.2021.1986920 10.1080/17482631.2021.1986920 PMC8547866 34694979

[15] Drugan J, (2017) Ethics and social responsibility in practice: interpreters and translators engaging with and beyond the professions, The Translator 23(2):126–142 10.1080/13556509.2017.1281204

[16] Roberts C, Darroch F, Giles A, Bruggen R (2022) You’re carrying so many people’s stories: vicarious trauma among fly-in fly-out mental health service providers in Canada, Int J Qual Stud Health Well-being 17(1) 10.1080/17482631.2022.2040089 10.1080/17482631.2022.2040089 PMC8925925 35195506

[17] Boudah DJ (2011) Conducting educational research: guide to completing a major project. Sage, London (Chapter 6)

[18] Braun V, Clarke V (2006) Using thematic analysis in psychology. Qual Res Psychol 3(2):77–101 10.1191/1478088706qp063oa

[19] Maguire, M. & Delahunt, B. 2017. Doing a thematic analysis: a practical, step-by-step guide for learning and teaching scholars. All Ireland J Higher Educ vol 9(3) 10.62707/aishej.v9i3.335

[20] Ashwin P, Boud D, Coate K, Hallett F, Keane E, Krause K, Leibowitz B, MacLaren I, McArthur J, McCune V, Tooher M (2015) Reflective teaching in higher education. London: Bloomsbury Publishing Plc, pp 16-260

[21] Costa DK, Moss M (2018) The cost of caring: emotion, burnout, and psychological distress in critical care clinicians. Ann Am Thorac Soc 15(7):787–790 10.1513/AnnalsATS.201804-269PS 29727197 10.1513/AnnalsATS.201804-269PS PMC6207112

[22] Lipscomb AE, Ashley W (2020) Surviving being Black and a clinician during a dual pandemic: personal and professional challenges in a disease and racial crisis. Smith Coll Stud Soc Work 90(4):221–236 10.1080/00377317.2020.1834489

